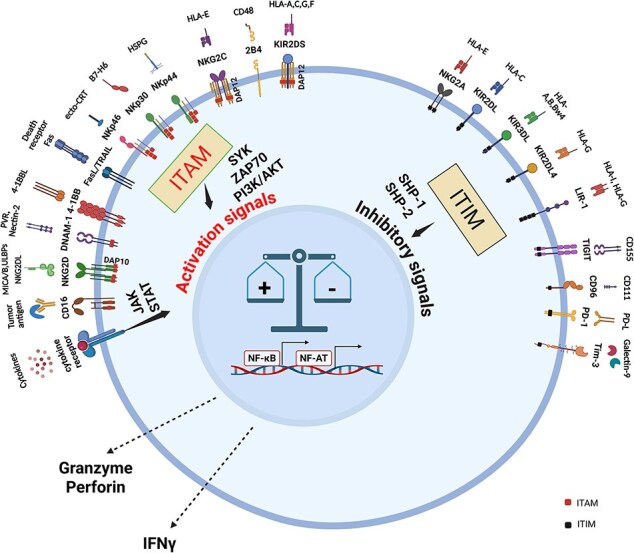# Correction to: CAR NK cell therapy for solid tumors: potential and challenges

**DOI:** 10.1093/abt/tbag004

**Published:** 2026-02-02

Correction to:

Yanlin Yu, Mitchell Ho, CAR NK cell therapy for solid tumors: potential and challenges, *Antibody Therapeutics*, Volume 8, Issue 4, October 2025, Pages 275–289, https://doi.org/10.1093/abt/tbaf019

The originally published version of this manuscript has been corrected to address an error in Figure 1.

Correction Details:

In the right-hand panel of Figure 1, the label “ITAM” was incorrectly used and has been changed to the correct term, “ITIM”.